# Fabrication of 3D-printed molds for polydimethylsiloxane-based microfluidic devices using a liquid crystal display-based vat photopolymerization process: printing quality, drug response and 3D invasion cell culture assays

**DOI:** 10.1038/s41378-023-00607-y

**Published:** 2023-11-09

**Authors:** Matthew D. Poskus, Tuo Wang, Yuxuan Deng, Sydney Borcherding, Jake Atkinson, Ioannis K. Zervantonakis

**Affiliations:** 1 Department of Bioengineering, UPMC Hillman Cancer Center, University of Pittsburgh, Pittsburgh, PA USA; 2 McGowan Institute of Regenerative Medicine, Pittsburgh, PA USA

**Keywords:** Other nanotechnology, Engineering

## Abstract

Microfluidic platforms enable more precise control of biological stimuli and environment dimensionality than conventional macroscale cell-based assays; however, long fabrication times and high-cost specialized equipment limit the widespread adoption of microfluidic technologies. Recent improvements in vat photopolymerization three-dimensional (3D) printing technologies such as liquid crystal display (LCD) printing offer rapid prototyping and a cost-effective solution to microfluidic fabrication. Limited information is available about how 3D printing parameters and resin cytocompatibility impact the performance of 3D-printed molds for the fabrication of polydimethylsiloxane (PDMS)-based microfluidic platforms for cellular studies. Using a low-cost, commercially available LCD-based 3D printer, we assessed the cytocompatibility of several resins, optimized fabrication parameters, and characterized the minimum feature size. We evaluated the response to both cytotoxic chemotherapy and targeted kinase therapies in microfluidic devices fabricated using our 3D-printed molds and demonstrated the establishment of flow-based concentration gradients. Furthermore, we monitored real-time cancer cell and fibroblast migration in a 3D matrix environment that was dependent on environmental signals. These results demonstrate how vat photopolymerization LCD-based fabrication can accelerate the prototyping of microfluidic platforms with increased accessibility and resolution for PDMS-based cell culture assays.

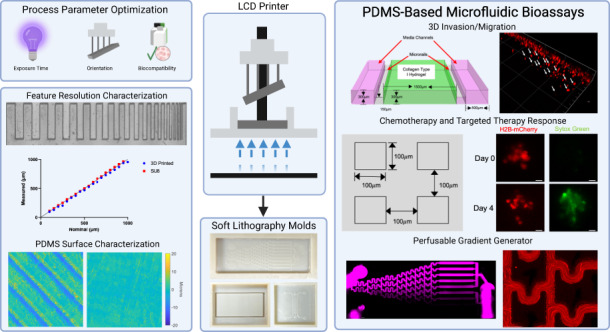

## Introduction

Microfluidic devices are characterized by their submillimeter (<1000 μm) features and fluidic channels^1^. Due to their high sensitivity, small reagent volumes^2^, and control of the cellular environment^3^, microfluidic devices are widely used in biological applications, such as cell migration studies^4^, drug sensitivity^5^, and angiogenesis^6^. Despite these benefits, microfluidic devices have yet to be widely adopted for biomedical research, in part due to high cost^7^ and fabrication difficulties, which create barriers to entry that may make the technology impractical for researchers to easily implement^8^. Standard microfluidic device fabrication using SU-8 soft lithography can be time-consuming (hours to days)^3,9^ and requires specialized training and facilities to fabricate molds^10,11^.

Additive manufacturing is a promising recent technology in the biomedical field that has several advantages over standard microfluidic fabrication methods^12–15^. Specifically, 3D printing has lower cost and fabrication time than SU-8 soft lithography^16,17^ and does not require a cleanroom^18^. These advantages make 3D printing suitable for the rapid prototyping of microfluidic designs^19,20^. 3D printing offers greater design flexibility than SU-8 soft lithography by permitting the formation of truly 3D structures rather than the planar geometries typical of photolithography^21,22^. Furthermore, it allows users to directly print microfluidic devices, generate molds for another fabrication material^18^, or augment existing cell culture platforms to precisely control environmental factors^23–25^. Vat photopolymerization is a promising 3D printing technology that offers greater resolution (18–250 μm resolution)^26,27^ and improved surface quality compared to fused deposition modeling 3D printing methods^13,16,28^. Specifically, a subset of vat photopolymerization processes allows direct printing of a 2D layer by using either a digital light projection (DLP) or liquid crystal display (LCD) screen as a light source to project a 2D image onto a photopolymerizing resin, locally curing a layer of resin in these illuminated regions. The part is formed layer-by-layer on a build stage until the completed 3D structure is formed^9,29^.

Current challenges with this technology include optimizing printer resolution and resin toxicity, as uncured resin components may be cytotoxic^30–32^. Commercial printer resolution is also a limiting factor for many researchers^33^. While advances have been made in analyzing the physical limitations and biocompatibility of resins^28,29,34,35^, few studies have characterized the performance of microfluidic devices made from polydimethylsiloxane (PDMS), a ubiquitous material used in biomedical applications^36^, fabricated via soft lithography using 3D-printed molds. Our work addresses these challenges by systematically characterizing the impact of both LCD-based vat photopolymerization printing protocols and resin on PDMS microfluidic feature resolution and cell viability. Herein, we demonstrate the impact of universally relevant printing parameters (layer height, part orientation, and exposure time) in optimizing the fabrication process and identifying feature resolution limits for multiple geometries. We utilize three relevant microfluidic geometries, microwells, gradient generators, and hydrogel-carrying devices, to evaluate the capabilities of a low-cost, commercially available 3D printer in cell viability, drug response, and 3D cell invasion assays. Cell viability in devices fabricated using 3D-printed molds is comparable to that in SU-8 devices, and sensitivity to cytotoxic chemotherapy is comparable to that in standard tissue culture plate assays. Furthermore, we demonstrate the generation of fluid flow-based concentration gradients in these microfabricated devices and monitor the dynamics of cancer cell invasion in a 3D matrix environment. In summary, we believe that these advances in PDMS device fabrication using low-cost, commercial LCD-based printers and the identification of biocompatible resins will accelerate the development of widely accessible microfluidic cell culture assays.

## Methods

### 3D-printed mold fabrication

Computer-aided design (CAD) models for all molds were generated using Autodesk Inventor (Autodesk, USA) and directly imported into the 3D printer slicer program Chitubox (Chitubox, China). Resin-specific default printing profiles were used unless otherwise specified. “Medium” support settings were used, and supports were automatically generated using the “+All” setting. Components were printed using an LCD-based Phrozen Sonic Mini 4 K (Phrozen Technology, Taiwan) resin printer with a screen protector (BulletBrandCompany, USA). Z-Calibration of the printer was performed per the manufacturer’s instructions prior to each print. Resin (Phrozen Technology, Taiwan) was filled to approximately 1/3 the height of the resin vat. After printing was completed, excess resin was removed from the components using compressed air. The unused resin was filtered using a 150 μm paper strainer (Shanqian, China) to be reused. Components were placed in an ANYCUBIC Wash and Cure station (Anycubic, China) to wash for 10 min. After washing, parts were removed from the printing stage using a metal spatula and placed in a plastic bag filled with 70% isopropanol/30% deionized (DI) water. The bag was placed in an ultrasonic cleaner (Kaimashi, China) and sonicated in a water bath for five minutes. The parts were subsequently dried with compressed air before placement in the ANYCUBIC Wash and Cure Station to cure for 60 min. The excess resin was removed from the building plate, and the build area was resurfaced by sanding using 60-grit sandpaper for 10–20 seconds prior to printing again. After curing, the parts were placed in an oven (Hybaid, USA) for 48 h before returning to storage at room temperature.

### PDMS device fabrication

Sylgard 184 PDMS was mixed in a 10:1 ratio elastomer base to curing agent by mass and placed in a vacuum desiccator for 1 h to remove bubbles. PDMS was poured into 3D-printed molds and placed overnight in an oven (Hybaid, USA) to cure before use. PDMS was removed from molds using a hobby knife. Debris was removed from devices prior to use with tape (Scotch, USA). The first pour of PDMS from all molds was discarded to account for the transfer of any potential uncured resin to PDMS.

### Cell culture

Breast cancer cell lines BT474 and MDA-MB-231 were generously provided by Dr. Dennis Slamon, University of California Los Angeles, Los Angeles, CA. BT474 and MDA-MB-231 cell lines, EFM192, and AR22 cell lines were engineered to express fluorescent nuclei (H2B-mCherry, H2BRFP, or H2BGFP) to enable single-cell tracking via fluorescence microscopy for quantification of viability, migration, and infiltration. Cells were subsequently sorted using fluorescence-activated cell sorting (FACS) to select for mCherry+, RFP+, or GFP+ cells. Tumor cells were grown in Roswell Park Memorial Institute (RPMI) 1640 medium (Corning, USA) supplemented with 10% heat-inactivated fetal bovine serum (FBS) (Avantador, USA) and 1% penicillin (100 units/mL)/streptomycin (100 μg/mL) (Gibco, USA). AR22 mammary fibroblasts were cultured in Dulbecco’s modified Eagle’s medium (DMEM) (Corning, USA) supplemented with 10% heat-inactivated FBS (Avantador, USA) and 1% penicillin (100 units/mL)/streptomycin (100 μg/mL) (Gibco, USA). Cells were cultured in a humidified incubator at 5% CO_2_ and 37 °C.

### PDMS microwell viability assay

Microwell 3D-printed molds for the initial cell viability experiments were fabricated using resin manufacturer-recommended printing parameters (50 μm layer height) and 55° orientation, where orientation refers to rotation of the mold about the x-axis on the build plate (Supplementary Fig. 1a). This orientation improved the successful print rate compared to 0° rotation and was calculated to be the printing angle at which aliasing was minimized for this layer height (Supplementary Fig. 1b). PDMS was sterilized by autoclaving (30 min at 121 °C wet cycle followed by 30 min at 121 °C gravity cycle). All molds were plasma treated using a plasma cleaner (Harrick Plasma, USA) to enhance surface hydrophilicity, which prevents air bubble formation in microwells. A reservoir for SU-8 microwells was created by plasma binding a second layer of PDMS to the surface of the microwells. Photolithography was used to produce micropatterned SU-8 molds (MicroChem, USA). Cells were collected by trypsinization (0.05% trypsin, Corning, USA) and seeded at 33,000 cells/cm^2^ in 1.75 mL for 3D-printed microwells, 500 μL in SU-8 microwells, and 200 μL in black 96-well plates (Greiner Bio-One, Germany). To monitor cell death, cells were cultured in medium containing 100 nM Sytox Green (Invitrogen, USA). Moreover, 100 nM Paclitaxel (Selleck Chemical LLC, USA) was added upon cell seeding in treatment conditions. Medium was replenished daily as needed. Imaging was performed using a Nikon Ti2 microscope (Nikon, Japan) equipped with a live-cell imaging stage (Tokai Hit, Japan). Analysis of fluorescence images was performed using an Ilastik^37^ machine learning pipeline and CellProfiler^38^ to classify individual cells as alive or dead. Viability was calculated as the fraction of live cells among total cells (alive plus dead). Replicates represent individual biological replicates. Error bars represent the standard error of the mean (SEM). Statistical analysis was performed using Student’s *t* test.

### Printing parameter analysis and minimum feature size

We systematically tested the effects of layer height, mold orientation, and exposure time on printing quality using microwells. PDMS poured from molds with different printing parameters was imaged via widefield microscopy using a Nikon Ti-2 (Nikon, USA). To determine the optimum printing parameters, the microwell aspect ratio was computed as the ratio of height to width using ImageJ. Replicates represent randomly sampled microwells.

Optical profilometry was performed using a Zeiss LSM700 (Carl Zeiss, Germany) confocal microscope. Optical sections of reflected light for 3D surface reconstruction from PDMS parts cast in molds without any features (flat surface) at the prescribed printer settings were acquired. The Z section interval was set at 2 μm intervals using a 20X objective (NA 0.8) to capture the surface profile of a x-y, 320 × 320 μm field for three random regions of PDMS cured in each mold. Surface reconstruction was performed by calculating the z-position at which the intensity of reflected light was maximal among all z-stacks for each pixel. Surface root mean square (RMS) values were calculated using MATLAB (MathWorks, USA).

For feature size characterization, 3D-printed molds were fabricated using the optimum 10 μm layer height and 29° two-axis rotation orientation settings, where two-axis orientation refers to rotation of the mold about the x- and y-axis on the build plate (Supplementary Fig. 1a). SU-8 wafers were fabricated in-house (University of Pittsburgh, USA). PDMS cast from molds was plasma treated and then bonded to a glass slide for imaging (Harrick Plasma, USA) with a Nikon Ti2 (Nikon, Japan) microscope. ImageJ was used to quantify the length of each side of the microwells. The mean side length for all edges is compared to the nominal feature side from the corresponding CAD model. Error bars represent SEM. Statistical analysis was performed using Student’s *t* test for the comparison of two groups or one-way ANOVA followed by Tukey’s multiple comparisons test for more than two groups.

### Gradient generator assay

Inlet and outlet ports in the device were punched using a 2 mm biopsy punch (Miltex, USA). The devices were attached to a 24 × 60 mm #1 coverglass (VWR, USA) via plasma treatment using a plasma cleaner (Harrick Plasma, USA). A solution of DI water or DI water containing 4 μg/mL 10 kDa Alexa Fluor-647-conjugated dextran (Invitrogen, USA) and a 1:20,000 dilution of red fluorescent 1 μm carboxylate-modified microspheres (Invitrogen, USA) was prepared. These solutions were aspirated into 10 mL syringes (BD, Switzerland) connected to microbore tubing (Masterflex, USA) that were connected to the microfluidic device via luer connectors (Qosina, USA). The outlet port was connected to tubing that fed into a waste beaker. Tubing was directly inserted into inlet/outlet ports to form a leakproof connection. Syringe pump-driven flow was established using a two-channel syringe pump (Harvard Apparatus, USA) at the prescribed flow rate. Flow was established for seconds to minutes depending on the flow rate to achieve a steady state for each condition prior to imaging. Imaging was performed on a Nikon Ti2 (Nikon, Japan) microscope. Channel intensity was quantified using NIS-Elements (Nikon, Japan), and ImageJ was used to measure the mean intensity in each channel.

### 3D invasion assay

Ports in the device were punched using a biopsy punch (Miltex, USA). The devices were sterilized via autoclave (30 min at 121 °C gravity cycle) and then attached to a 22 × 40 mm #1 coverglass (VWR, USA) via plasma treatment using a plasma cleaner (Harrick Plasma, USA). Once bound, devices were incubated at 80 °C for 48 h to return the PDMS surfaces to a hydrophobic state. The central channel of the devices was filled with buffered collagen type I (Corning, USA) at a concentration of 1 mg/mL and incubated for 30 min at 37 °C for polymerization. MDA-MB-231 breast cancer cells were subsequently seeded at a concentration of 0.5 × 10^6^ cells/mL into the device (40 μL) in either 0% FBS medium (starvation) or 10% FBS medium (complete medium). The devices were placed on an incubator stage (Tokai Hit, Japan) for live-cell imaging using a Nikon Ti2 microscope (Nikon, Japan). Cells were imaged every 15 min for 8 h. The devices were fixed using 4% paraformaldehyde (Electron Microscopy Sciences, USA) after 24 h. Cell invasion analysis was performed using ImageJ TrackMate^39^. Confocal imaging was performed using a Zeiss LSM700 (Carl Zeiss, Germany) with a 5 μm z-step. Data are representative of at least two biological replicates. Error bars represent SEM. Statistical analysis was performed using Student’s *t* test.

### Microfluidic coculture assay

Ports in the device were punched using a biopsy punch (Miltex, USA). The devices were sterilized via autoclave (30 min at 121 °C gravity cycle) and then attached to a 22 × 40 mm #1 coverglass (VWR, USA) via plasma treatment using a plasma cleaner (Harrick Plasma, USA). Once attached, the devices were incubated at 80 °C for 48 h to return the PDMS surfaces to a hydrophobic state. The central channel of the devices was filled with buffered collagen type I (Corning, USA) at a concentration of 2 mg/mL. Either EFM192-H2BRFP (monoculture) or EFM192-H2BRFP and AR22-H2BGFP (coculture) were added, each at a final concentration of 0.5 10^6^ cells/mL in the collagen gel. Devices were cultured in RPMI medium containing either DMSO (control) or 1 µM lapatinib and 250 nM Incucyte Cytotox NIR dye (Sartorius, USA) to selectively stain for dead cells. Devices were imaged via confocal microscopy daily for 48 h. Viability was calculated as the fraction of alive to total cells (dead plus alive). Data are representative of at least two biological replicates. Error bars represent SEM. Statistical analysis was performed using one-way ANOVA followed by Tukey’s multiple comparisons test.

### Microfluidic Coculture Staining

Staining was performed on fixed samples permeabilized with 0.1% Triton-X (Sigma Aldrich). Samples were incubated with blocking buffer for 1 h, stained with phospho-S6 (Cell Signaling Technology #5364) or Ki67 (Abcam 15580) unconjugated antibody (1:100 dilution), and incubated overnight. Samples were washed 3× with PBS prior to staining with secondary antibodies (Invitrogen A-31556) (1:200 dilution) for 2 h. Images were acquired via confocal microscopy. Data are representative of at least two biological replicates. Statistical analysis was performed using one-way ANOVA followed by Tukey’s multiple comparisons test.

### Spheroid coculture assay

Microwell array molds (300 × 300 µm) were printed using a 3D printer. Patterned PDMS was cut into disks with the diameter of one microwell of a 96-well plate and autoclaved. PDMS was placed in each well of a 96-well plate (Greiner Bio-One, Germany), and the plate was plasma-treated prior to seeding cells. EFM192-H2BRFP (monoculture) or EFM192-H2BRFP and AR22-H2BGFP (coculture) cells were seeded at a density of 10,000 cells/well in 100 μL of the medium. Immediately after seeding, plates were centrifuged at 900 RPM for 3 min to force cells into the microwells. After 72 h, cultures were dosed with 100 μL of either control or treatment (1 μm final concentration lapatinib) medium containing 250 μm Incucyte Cytotox NIR. Images were acquired via confocal microscopy every day for 96 h. Viability was calculated as the percentage of live-cell area versus total cell area. Data are representative of at least two biological replicates. Error bars represent SEM. Statistical analysis was performed using one-way ANOVA followed by Tukey’s multiple comparisons test.

### Fibroblast microfluidic migration assay

Ports in the device were punched using a biopsy punch (Miltex, USA). The devices were sterilized via autoclave (30 min at 121 °C gravity cycle) and then attached to a 22 × 40 mm #1 coverglass (VWR, USA) via plasma treatment using a plasma cleaner (Harrick Plasma, USA). Once attached, the devices were incubated at 80 °C for 48 h to return the PDMS surfaces to a hydrophobic state. The central channel of the devices was filled with buffered collagen type I (Corning, USA) at a concentration of 2 mg/mL. Either AR22-H2BGFP (monoculture) or EFM192-H2BRFP and AR22-H2BGFP (coculture) were added at a final concentration of 0.5 × 10^6^ cells/mL in the collagen gel. Cells were treated with a control medium or 1 μm lapatinib for 48 h. After 48 h, the devices were imaged every hour for 4 h. Cell trajectories were computed using TrackMate. Data are representative of at least two biological replicates. Statistical analysis was performed using one-way ANOVA followed by Tukey’s multiple comparisons test.

### Two-gel interface design

Ports in the device were punched using a biopsy punch (Miltex, USA). The devices were sterilized via autoclave (30 min at 121 °C gravity cycle) and attached to a 22 × 40 mm #1 coverglass (VWR, USA) via plasma treatment using a plasma cleaner (Harrick Plasma, USA). Once attached, the devices were incubated at 80 °C for 48 h to return the PDMS surfaces to a hydrophobic state. The two-gel channels of the devices were filled with buffered collagen type I (Corning, USA) at a concentration of 2 mg/mL. EFM192-H2BRFP and AR22-H2BGFP were added, each at a final concentration of 0.5 × 10^6^ cells/mL in the collagen gel. Images were acquired via confocal microscopy.

### Statistical analysis

Statistical analysis was performed using GraphPad Prism (GraphPad, USA). A *p* value of <0.05 was considered to indicate statistical significance. The number of replicates and what the error bars represent are described in the legend for each figure and in the methods section.

## Results

### Fabrication of printed molds for PDMS casting using LCD-based 3D printing

**Fig. 1:**
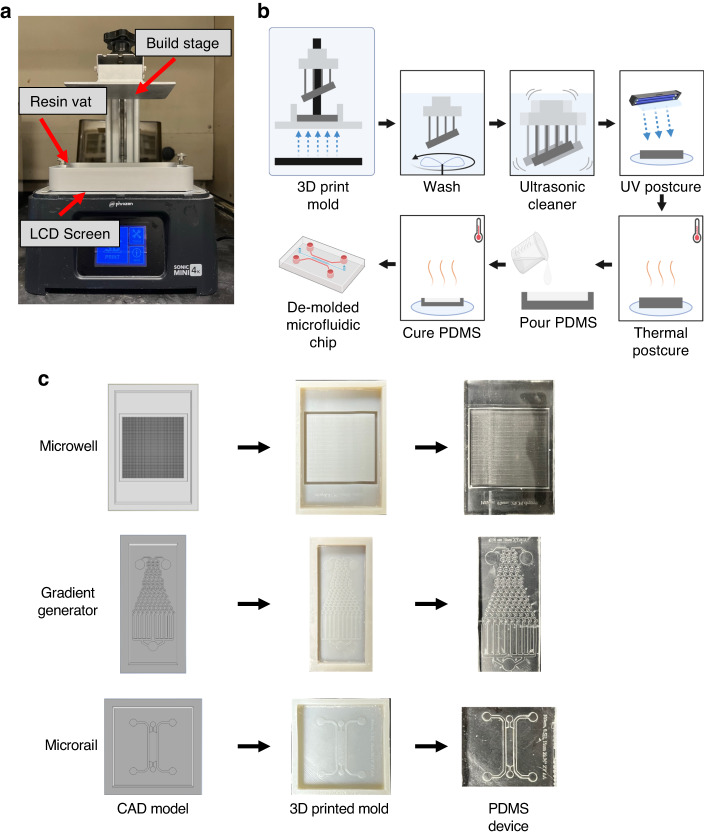
**Process overview for vat photopolymerization 3D printing of microfluidic devices.** **a** Commercially available Phrozen Sonic Mini 4 K printer used for mold fabrication. **b** Workflow for fabrication of microfluidic devices using LCD 3D Printing. **c** Representative images of CAD model (left), 3D-printed mold (middle), and PDMS microfluidic chip (right) for geometries tested

A commercially available LCD-based Phrozen Sonic Mini 4 K resin printer (Fig. 1a) was used to fabricate 3D-printed molds. Briefly, CAD models (i.e., STEP files) are imported into Chitubox slicer software for conversion to 3D printer format and to set 3D printing parameters (e.g., layer height, exposure time, supports, etc.). Printing is performed by loading the slicer file onto the printer via USB drive and filling resin vat. The LCD screen below the resin vat illuminates specific pixels to project an image of the slice onto the resin to induce local photopolymerization of the resin to form one cured layer. This process is repeated for each layer. After curing, the printed parts undergo a series of postprocessing steps, including two washing steps, a light-exposure postcure, and a thermal postcure, to prepare the mold for soft lithography and to limit potential PDMS curing inhibition (Fig. 1b). PDMS is poured into the molds to produce microfluidic devices (Fig. 1c).

### Cell viability is not dependent on resin, and drug response in microwells reflects tissue culture plate results

**Fig. 2:**
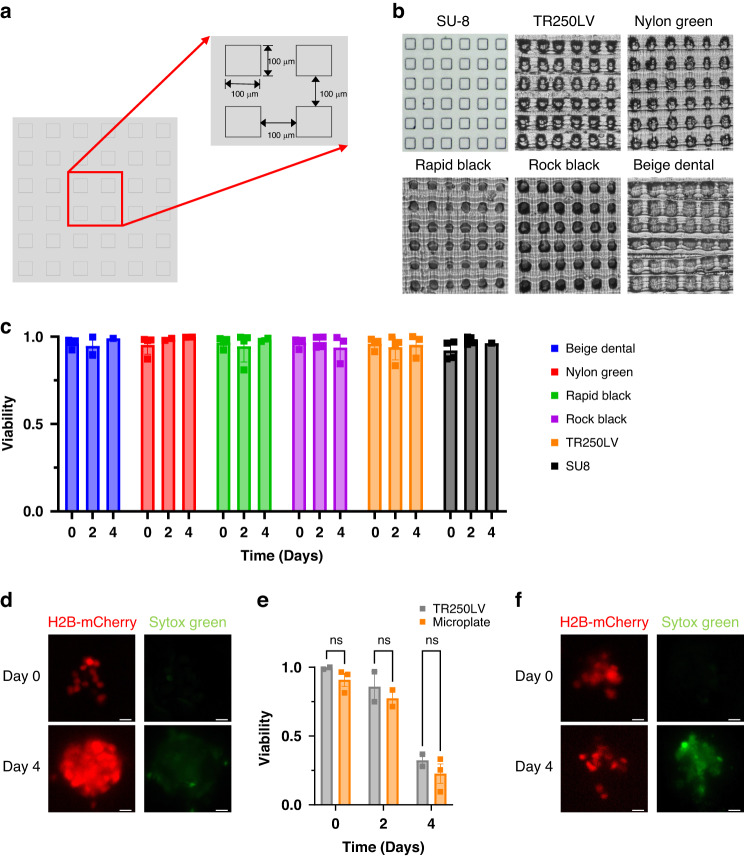
Commercial resins do not impact cell viability compared to standard photolithography-based microwells and drug response in 3D printed microwells reflects traditional microplate results. **a** Annotated CAD model of microwell array dimensions. **b** Representative images of PDMS from 3D-printed molds for tested resins. **c** Viability after 4 days of cancer cells seeded in PDMS from 3D-printed molds. **d** Representative images of tumor cells (red) seeded in microwells after 4 days. Green represents dead cells. **e** Viability after 4 days of cancer cells seeded in PDMS of treatment with 100 nM paclitaxel. Statistical analysis performed using Student’s *t* test. Data are representative of at least two independent biological replicates. **f** Representative images of tumor cells seeded in microwells after paclitaxel treatment Green represents dead cells. Scale bars represent 25 μm. Error bars denote SEM

We identified several commercially available resins that may be suitable for 3D printing molds for soft lithography. We limited our selection to resins recommended for our printer, as we reasoned that these resins would have the greatest performance without exhaustive testing and validation. We selected five resins with a range of mechanical properties for our studies (beige dental, nylon green, rapid black, rock black, TR250LV Table 1). To assess the performance of these resins, we first compared the printed quality of an array of 100 × 100 μm microwells fabricated from 3D-printed molds to the SU-8 gold standard (Fig. 2a). Each resin was able to produce distinct microwell features (Fig. 2b). We noted that the beige dental resin developed cracks in the mold after the thermal postcure stage (Supplementary Fig. 2a, b). We next seeded BT474 tumor cells into 3D-printed microwells to assess the biocompatibility of PDMS from each of the resins for up to four days. The viability of tumor cells in microwells exceeded 85% viability for all resins (Fig. 2c, d). We next evaluated the cell response to cytotoxic chemotherapy. We treated BT474 tumor cells with 100 nM paclitaxel for 4 days in the microwells fabricated using the 3D-printed molds and in a parallel experiment in tissue culture plates (Fig. 2e, f). We found that the drug response was similar between the standard plate and our 3D-printed microwell assay, as measured up to four days after treatment. These results indicate that the tested resins produce biocompatible microfluidics that mimic the drug response of conventional cell culture systems.

**Table 1:** Comparison of mechanical and thermal properties of tested resins

Resina	Viscosity (cps)	Ultimate tensile strength (MPa)	Tensile modulus	Heat deflection temperature (°C)
Nylon green	850–905	24	600	Not specified
TR250LV	180–280	25	900	100–120
Rock black	70–170	30	419	97
Rapid black	70–170	15	110	Not specified
Beige dental	700–800	Not specified	Not Specified	Not specified

### Fabrication process optimization and characterization of minimum feature size

To systematically characterize the impact of each process parameter on printing quality, the TR250LV resin was selected owing to its heat deflection temperature and mechanical properties^40^ (Table 1). As the biocompatibility of PDMS devices fabricated using all resins seemed comparable, we reasoned that these properties may increase mold longevity after repeated use and heat cycling when curing PDMS. We first explored the impact of layer height (for a fixed print orientation at 55°) on printed device quality by examining microwells of PDMS cast from molds printed at 50 μm and 10 μm. PDMS devices fabricated from molds printed at 10 μm had fewer and less severe aliasing lines (Supplementary Fig. 3a) than molds printed at 50 μm. Furthermore, the aspect ratio, measured as the ratio of length to height of individual microwells, was significantly closer to the nominal value of unity for the designed microwells printed with a 10 μm layer height (Supplementary Fig. 3b). Therefore, we continued all future prints with a 10 μm layer height. The impact of print orientation was next explored by evaluating the effects of the angle and the number of rotations either along the x-axis or both the x- and y-axes (Supplementary Fig. 1a). First, molds were printed at different angles of 55°, 29°, or 15°. Printing at 0° (flat printing) often resulted in print failure, so this orientation was not considered. These angles were selected to vary the extent of aliasing by integer numbers of voxels at the surface of the printed mold. These angles create a surface stairstep pattern of 5, 2, or 1 vertically stacked 35x35x10 μm voxels for every 1 horizontal voxel. The x- and y-dimensions correspond to the pixel size (35 μm), and the height corresponds to the layer height (10 μm) (Supplementary Fig. 1b). PDMS cast from molds printed at these angles was examined. At the 29° and 15° orientations, aliasing was reduced compared to the 55° orientation. However, at shallower printing angles, the microwells became elongated rather than square and had an increased aspect ratio. Since both the 29° and 15° microwells had minimal aliasing compared to the 55° microwells, the 29° angle was considered superior to the 15° angle due to its lower elongation (Supplementary Fig. 3d). However, we also observed a consistent surface distortion in the center of these 3D-printed molds printed at 55°, 29°, and 15°. To correct this, we oriented molds after rotating about both axes (the x- and y-axes) rather than one (the x axis) (Supplementary Fig. 3e). This resulted in increased shape distortion of microwells (Supplementary Fig. 3f) but less elongation (Supplementary Fig. 3g). Finally, the impact of layer exposure time on PDMS cast from molds was examined. At an exposure time below 2.5 s, the microwells were undeveloped (Supplementary Fig. 3h). These analyses identified optimal printing settings: 10 μm layer height, 29° orientation along two axes, and a 2.5 s exposure time for the TR250LV resin. We examined the shape of PDMS from 3D-printed molds with these optimized settings versus that from SU-8 molds to characterize the microwell area and well-to-well variation obtained using 3D-printed and SU-8 molds (Supplementary Fig. 3i).

To further understand how printing process parameters influence the quality of PDMS from 3D-printed molds, we utilized confocal optical profilometry to interrogate how the PDMS surface profile is affected by layer height and print orientation. To determine whether these observations are consistent across resins, we measured the surface of TR250LV, beige dental, and green nylon resins. First, we examined the surface profile RMS, a metric for surface roughness, where a lower surface RMS indicates that the surface is smoother than a surface with a higher RMS (Supplementary Fig. 4a). We first compared the molds printed at 50 μm and 10 μm layer heights. Across all resins, the surface RMS was lower for the molds printed at 10 μm than for the molds printed at 50 μm (Supplementary Fig. 4b). Next, we assessed how the print orientation affects the printed surfaces. We printed molds at 55°, 29°, and 15° and observed a trend in which the surface RMS decreased with shallower printing angles (Supplementary Fig. 4c). Finally, we evaluated RMS for prints oriented along one axis compared to two axes and found that two-axis printing reduced the surface RMS compared to single-axis printing (Supplementary Fig. 4d).

**Fig. 3:**
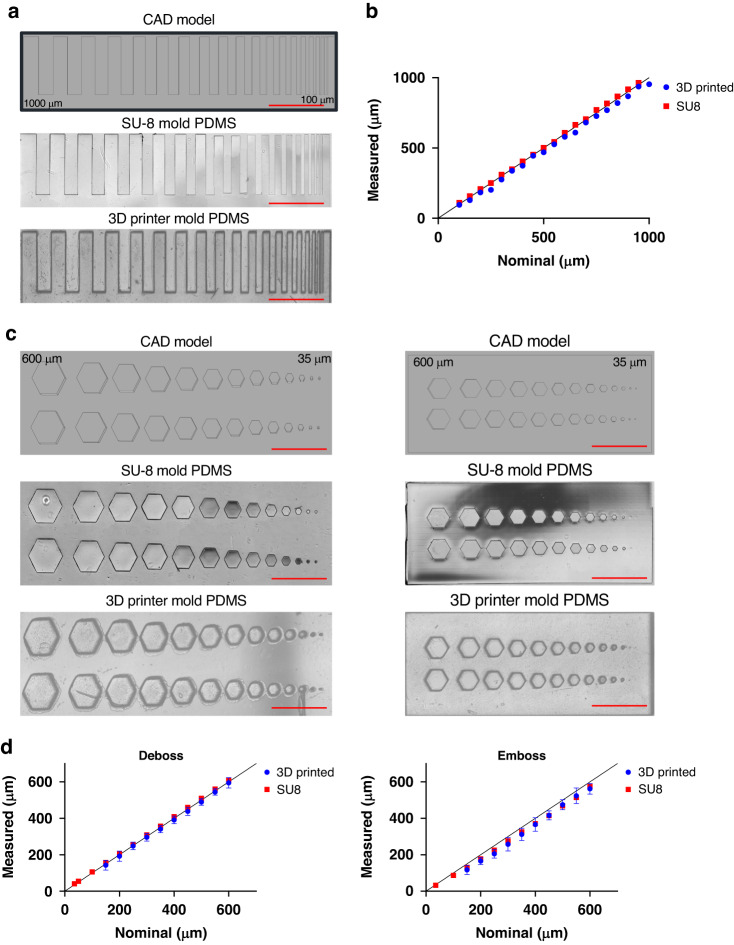
**Minimum feature size of LCD 3D-Printed microfluidics.** **a** Representative images of lines of varying width. **b** Quantification of nominal vs. measured line width of PDMS from SU-8 and 3D-printed molds. **c** Representative images of debossed (left) and embossed (right) hexagons. **d** Quantification of feature size for debossed (left) and embossed (right) hexagons. Scale bars represent 1 mm. Error bars denote SEM and represent individual length measurements (*n* = 6 sides) for two features

We next determined the minimum feature size that can be resolved by our printer given the optimum printing parameters. To this end, we printed positive and negative features of varying shapes and sizes and compared the measured and nominal feature sizes. First, a series of rectangles of decreasing width were printed and measured using ImageJ (Fig. 3a). The measured feature size closely matched the nominal feature size (Fig. 3b). All lines down to 100 μm were resolved in the fabricated PDMS devices using the 3D-printed molds. Next, we evaluated the minimum feature size using hexagonal embossed and debossed features. 3D-printed molds were fabricated, and the feature size in the PDMS-casted devices was compared to the nominal feature size (Fig. 3c, d) ranging from 35 to 600 μm in the length of each side. While all features were resolved in the SU-8 PDMS, only hexagons with lengths down to 150 μm were sufficiently resolved for measurement (Fig. 3c, d). Together, these results define the functional resolution capabilities of the LCD printer under the optimized printing parameters.

### Flow in microchannels and generation of concentration gradients

**Fig. 4:**
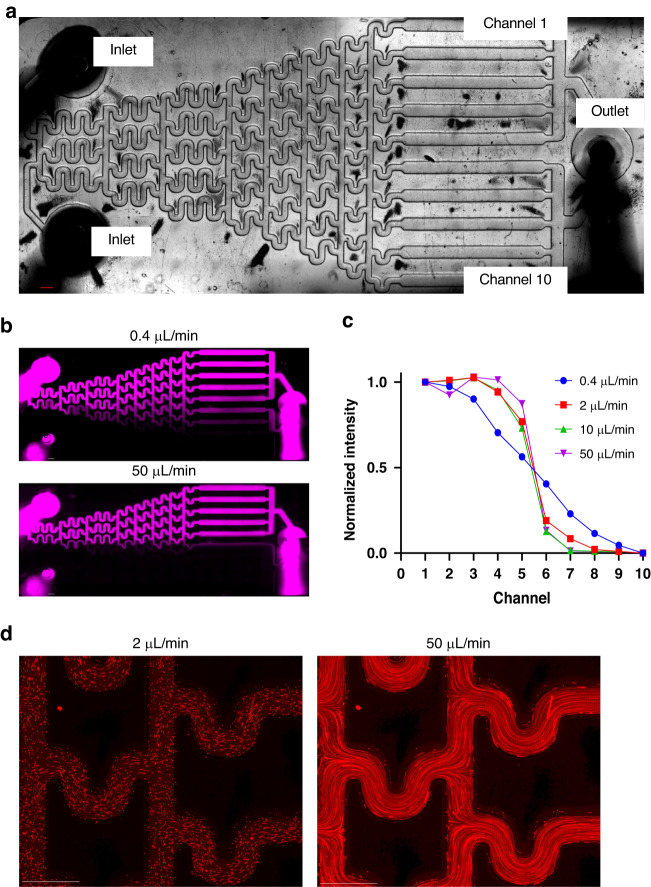
**Microfluidic gradient generator fabricated using LCD 3D printing.** **a** Schematic of microfluidic gradient generator. **b** Dextran gradient produced by perfusing at 0.4 μL/min (top) and 50 μL/min (bottom). **c** Intensity of fluorescent dextran measured in each channel for tested flow rates. **d** Fluorescent beads flowing through gradient generator at 2 μL/min (left) and 50 μL/min (right). Scale bars represent 1 mm

We employed a Christmas-tree microfluidic gradient generator to demonstrate the establishment of flow in microchannels and concentration gradients in PDMS-based devices fabricated using 3D-printed molds. This microfluidic concentration gradient generator consists of two inlets, one outlet, and ten channels, across which a concentration gradient is formed (Fig. 4a). A solution of fluorescently labeled (10 kDa Alexa Fluor-647) dextran and red fluorescent beads (1 μm) suspended in DI water was perfused through the top inlet and pure DI water in the other inlet to form a gradient. A two-channel programmable syringe pump was used to ensure identical flow rates into each inlet. Flow was established at 0.4–50 μL/min, and images of the device were acquired using fluorescence microscopy. At low flow rates, a gradual change in dextran concentration occurred across the device channels, whereas at high flow rates, a step change in concentration occurred (Fig. 4b, c). Fluorescent beads were used to probe particle streaklines within the device to ensure that channels did not leak. At both low and high flow rates, particle streaklines did not deviate from the channel, suggesting that the channels maintain strong adhesion to glass even at high flow rates (Fig. 4d). Therefore, these devices can maintain sustained perfusion even with complex gradient generator geometries.

### Real-time analysis of cancer cell invasion in a 3D collagen matrix

**Fig. 5:**
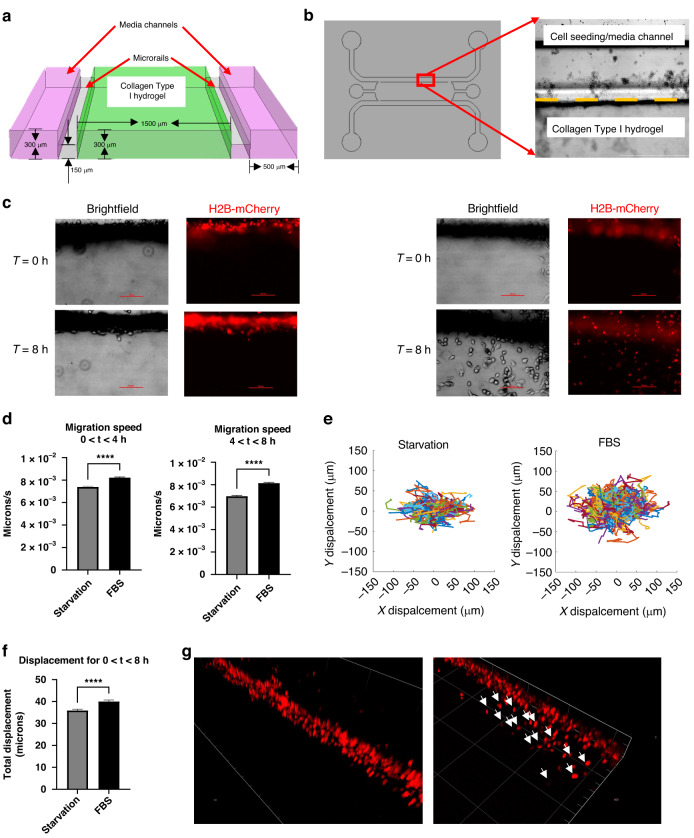
**Rail-based microfluidic invasion assay.** **a** Cross section of microchannels. Cells are seeded in collagen gel channel (green) which is separated from media channels (pink) by a narrow rail (gray) that constrains the collagen gel. **b** Gel-media interface seeded with MDA-MB-231 cancer cells. Dashed line indicates interface. **c** Representative images of MDA-MB-231 cancer cells (red) stimulated with starvation (no FBS, left) or FBS-containing full media (right) after 8 h. **d** Quantification of migration speed of single cells. Statistical analysis performed using Student’s *t* test. **e** Trajectories for starvation and FBS-containing conditions where each line represents the path of a single cell. **f** Quantification of cancer cell displacement after 8 h. Statistical analysis performed using Student’s *t* test. **g** Confocal imaging of invading cancer cells in starvation (left) or FBS-containing full media (right) conditions. White arrows indicate invading cancer cells. Data is representative of at least two independent biological replicates. Error bars denote SEM. *p < 0.05, ****p < 0.0001

A microfluidic cancer cell invasion assay was performed to assess device performance in sustaining embedded hydrogels and tracking cell invasion in 3D. The design incorporates a reduction in channel height from 300 μm of the medium channel to 150 μm of the 3D collagen type I hydrogel, which constrains the hydrogel through surface tension (Fig. 5a). Briefly, collagen was polymerized in the center microfluidic channel, and MDA-MB-231 breast cancer cells were seeded in the outer channel in either serum-starved or complete medium conditions (Fig. 5b). Cancer cells attached at the channel-gel interface and were imaged every 15 min to track individual cells for up to 8 h. After the first 8 h, cells invaded the gel in complete medium conditions but not starvation conditions (Fig. 5c). We tracked the migration trajectory of individual cells as they invaded and asked whether the migration speed varied between complete medium and starvation conditions. For the 0–4 and 4–8-hour windows, the migration speed of cells in complete medium was greater than that of cells under starvation conditions (Fig. 5d). Additionally, the total displacement of cells over 8 h was greater for cells in complete medium than in starvation medium (Fig. 5e, f). We also confirmed the distribution of invaded cells in the 3D collagen hydrogel via confocal imaging (Fig. 5g). These findings demonstrate that microfluidics fabricated using 3D printing can be used to monitor real-time cellular invasion of single cells.

### 3D coculture models to examine the impact of cell‒cell interactions on the targeted therapy response

**Fig. 6:**
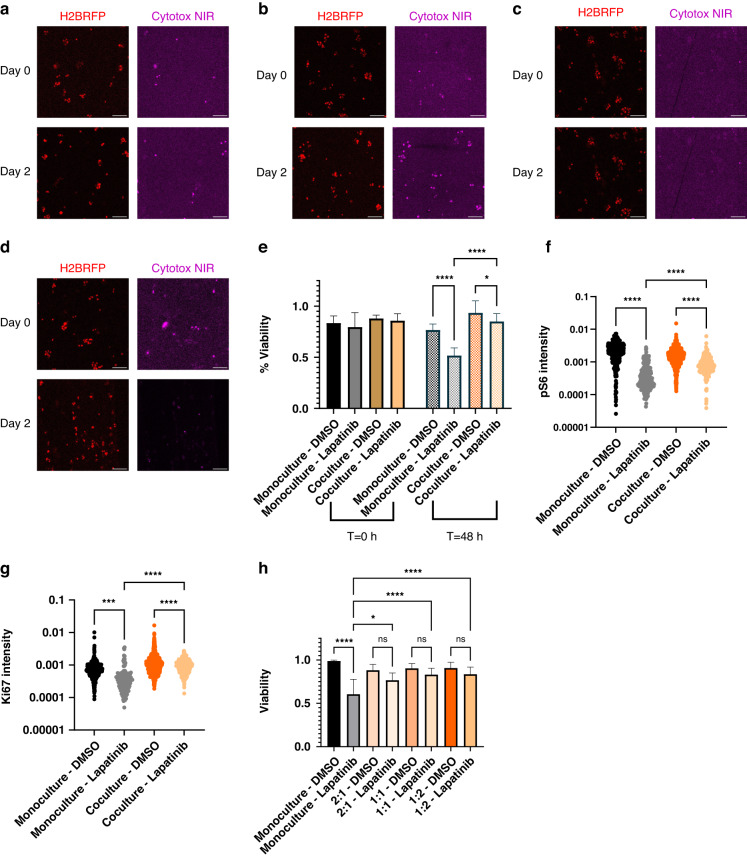
**Fibroblasts protect HER2+ breast cancer cells from the targeted therapy lapatinib.** **a** Representative images of EFM192 monoculture treated with DMSO or **b** lapatinib. Dead cells are indicated by Cytotox NIR+ staining. **c** Representative images of EFM192 cocultures treated with DMSO or **d** lapatinib. **e** Quantification of tumor cell viability. Error bars represent SEM of at least *n* = 3 individual devices. **f** Quantification of pS6 in tumor cells for at least *n* > 250 cells. **g** Quantification of Ki67 expression in tumor cells. **h** Quantification of tumor spheroid viability after 5 days of treatment for at least *n* > 250 cells. Error bars represent SEM of *n* = 8 replicate wells among two biological replicates. Scale bars represent 500 μm. Data are representative of at least two biological replicates. All statistical analysis was performed using one-way ANOVA followed by Tukey’s multiple comparison test, *p < 0.05, ****p < 0.0001

We next examined how cell‒cell interactions can impact drug response in two 3D assays. To this end, we cocultured EFM192 breast cancer cells with AR22 mammary fibroblasts, an abundant cell type in the breast tumor microenvironment^41^. We used cell seeding in a 3D collagen type I hydrogel and examined the drug response of tumor cells to the clinically relevant HER2-targeted therapy lapatinib^42^. After 48 h of treatment with lapatinib, a greater percentage of tumor cells survived when cocultured with fibroblasts than when grown in monoculture (Fig. 6a–e). These results are corroborated by the greater expression of phosho-S6 and Ki67 proteins, which are markers of survival signaling and cell cycle progression, respectively, in treated tumor cells under coculture conditions than in monocultures (Fig. 6f, g). Interestingly, we also found that the migration speed of fibroblasts was increased by the presence of either tumor cells or lapatinib (Supplementary Fig. 5a). As an orthogonal assay, we also assessed the drug response of tumor-only and tumor-fibroblast spheroids of varying tumor:fibroblast ratios ranging from 2:1 through 1:2 (Supplementary Fig. 5b–e). In agreement with our microfluidic assay, the presence of fibroblasts reduced the sensitivity of tumor spheroids to lapatinib after 96 h of treatment with lapatinib (Fig. 6h), where greater fibroblast density modestly increased cancer cell survival. To study the effects of heterotypic paracrine interactions, we proposed a novel, continuous hydrogel-hydrogel interface (Supplementary Fig. 5f) that simultaneously incorporates secreted factor crosstalk and monitoring of cell migration. As a demonstration of this design, we patterned EFM192-H2BRFP and AR22-H2BGFP in adjacent gel regions (Supplementary Fig. 5g).

## Discussion

Vat photopolymerization-based 3D printing is a relatively low-cost and rapid alternative to conventional SU-8 lithography techniques for microfluidics fabrication. However, the lack of standardized protocols for fabricating biocompatible microfluidics and the interdependency of fabrication parameters have hindered the adoption of this technology. To address this need, we established a pipeline to fabricate PDMS-based microfluidic devices using a low-cost (<$300) commercially available LCD-based 3D printer. First, we evaluated the cytocompatibility and chemotherapy-induced death dynamics in microfluidic devices fabricated using our 3D-printed molds. We systematically evaluated the effects of process parameters to maximize the resolution of 3D-printed parts and characterize the resolution limits. We subsequently fabricated devices that can maintain flow-induced concentration gradients or real-time single-cell tracking to monitor cell invasion in a 3D environment.

Previous studies directly used 3D-printed devices for cell culture rather than printing molds for soft lithography microfluidics^43,44^; however, direct printing has several potential challenges that require further investigation. Optical transparency in the UV‒Vis spectrum is crucial for fluorescence microscopy, as light must pass through the device to the detector. Light absorbed or scattered by the device may limit the amplitude of the signal that can be detected. The resins characterized in this study are opaque and therefore unsuitable for the imaging of directly printed microfluidic devices. Although optically clear resins are commercially available, some resins absorb light below 400 nm or are autofluorescent in common fluorescence imaging channels, such as DAPI and GFP^43,45,46^. Therefore, careful resin selection for direct printing is necessary when fluorescence imaging is to be performed. To further improve light transmission, several mechanical and chemical processes can be applied to printed parts, such as polishing and the application of thin resin, acrylate, or oil coatings^43,44^. However, these processes may affect the dimensions of the printed features^44^. In contrast, PDMS has high optical transparency in these spectra^47^.

Additionally, material stiffness critically impacts cellular phenotypes^48^. The stiffness of PDMS can be tuned by varying the composition, cure temperature, and cure duration to achieve stiffnesses between 0.8 and 10 MPa^49^. Conversely, the stiffness of the resins tested can be between 2 and 3 orders of magnitude greater^40^. A limitation of our soft lithography process is that the geometry of our cast microfluidic devices must permit removal from the printed mold. This effectively restricts the designs to open-channel designs that are sealed by covalently binding another material, such as cover glass, to the bottom surface. Although multilayer microfluidic devices^50^ provide greater design freedom, multiplane, variable-cross-section channels^51^ can only be achieved through direct printing. The combined benefits of both of these methods can be realized through the direct printing of PDMS, which takes advantage of the design freedom of direct printing with the ideal physical properties of PDMS^52^. Overall, the large variation in resin optical and mechanical properties as well as the limitations of soft lithography warrant the judicious selection of both resin and fabrication methods depending on the cell culture application.

We characterized the viability of cells cultured in PDMS microwells cast in five commercially available resins and found that all resins yielded devices that achieved high (<85%) viability. We believe that improved viability was achieved due to our indirect culture protocol using autoclaved PDMS devices with lower potential toxic compounds from resins. Consistent with this, previous studies have also demonstrated that cell viability in 3D-printed culture platforms can be improved via indirect culture and the soaking of 3D-printed molds in buffer^43^. On the other hand, direct cell culture on 3D-printed resins has been reported to impair cell viability and morphology^53^.

We next evaluated how printing process parameters such as layer height, print orientation, and exposure time affect the quality of 3D-printed microwells. We first examined the effects of layer height and found that reducing the layer height of microwells from 50 μm to 10 μm minimized t aliasing and coarseness in our printed microwells, which is consistent with other reports^54^. We next analyzed the influence of print orientation and found that a 29° orientation in the *x*- and *y* axes best balanced the observed aliasing and skewing. We reason that the high degree of skew observed with decreasing orientation angle may be attributed to greater bleed-through caused by a greater number of overhang layers. The number of layers that include an overhang formed from the printing of 100 × 100 × 100 μm cubes that form each microwell increases with shallower print angles. Since overhang layers can cause bleed-through by unwanted light penetration^43^, this bleed-through may distort individual microwells by overdeveloping the overhanging edge; however, this remains to be rigorously tested. We also determined the impact of exposure time by printing at two different exposure times. At the shorter exposure time, we noted that microwell features were highly deformed or not present at all. The reduced dose of light likely reduced the extent of polymerization below a critical threshold, as discussed by Gong et al., resulting in limited or no resin polymerization^55^.

Optical profilometry enables 3D reconstruction of a surface through measurement of light reflected by that surface^56^. This permits the interrogation of features at the printer voxel scale to elucidate mechanisms behind macroscale observations (e.g., greater separation forces between the part and the vat floor that may result in surface defects)^57^. Using optical profilometry, we evaluated the effects of layer height and print orientation (print angle and single- or double-axis rotation) on surface quality for three different resins (Supplementary Fig. 4). First, our observation that surface RMS decreased with layer height is consistent with previous reports that mathematically showed that the cusp height surface roughness is caused by the discretization of an angled surface into individual layers through layer-by-layer manufacturing, such as vat photopolymerization. Wang et al. showed that this metric is directly related to layer height, where a smaller layer height yields a lower cusp height^58,59^. Second, we explored how printing orientation affects surface quality. The equations derived by Wang et al. predict that the cusp height can be minimized by orientating the part surface such that each layer steps one voxel in the Y-direction and one voxel in the Z-direction (Supplementary Fig. 1b, left). This orientation angle (i.e., rotation angle from the horizontal about the *X* axis) is defined as the inverse tangent of the ratio of layer height to pixel size. Consistent with this prediction, we observed minimal surface RMS at the calculated optimal angle of 15° for our printing configuration (i.e., 10 μm layer height and 35 μm pixel size) and greater surface RMS at angles that greatly deviated from this value. Third, we compared the surface quality of molds printed on single-axis versus two-axis rotations and noted that the surface RMS was lower for molds printed on a two-axis orientation compared to a single-axis orientation. Pan et al. determined that the forces generated as a printed layer separate from the resin vat film are determined by the area-to-perimeter ratio of the printed surface^60^. As orienting the print along two axes reduces this value, the separation forces may be lower, and therefore, the extent of surface defects may be lower, resulting in a decreased surface RMS^57^.

After optimizing the printing parameters, we determined the minimum feature size created by our setup to be 100 μm and 150 μm for straight lines and hexagonal features, respectively. This is consistent with the minimum feature size reported by others using commercially available systems^43,51^. However, a limitation of the resolution of our system is that we cannot generate features small enough for single-cell applications (tens of microns scale). Furthermore, while our 3D-printed microwells can confine cells to form spheroids, the rounded edges of the well may be problematic for containing highly migratory cell types without distinctive edges. Improvements in resolution, such as using customized equipment for high-resolution, small-volume printing^27^, may resolve these issues. Other methods to improve resolution include altering the resin composition of the photoabsorber to reduce the extent of light penetration^55^ and tuning the wavelength of light emitted by the printer^51^.

Both LCD and DLP technologies cure a photosensitive resin layer-by-layer by forming an image of the layer to be printed on the resin vat surface. To generate the pattern, LCD printing selectively transmits light through pixels of an LCD screen, whereas DLP printing projects the image using individually maneuverable digital micromirror devices to either direct light toward (“ON” state) or away from (“OFF” state) the resin vat^61^. Commercial LCD printers were introduced following DLP printers and generally exhibit lower print quality^62^; however, they are typically cheaper for a given printer size due to a less complicated optics system^63^. The LCD screen absorbs a large amount of energy, resulting in lower energy output than DLP systems^64^, which impacts resin formulation for each printer type. Both DLP^48^ and LCD systems^47^ can generate intermediate light intensities; however, LCD printing does not suffer from distortions caused by the lenses and mirrors present in DLP systems^63^. Due to weak light leakage through the crystals in the LCD screen, the precision of LCD-printed parts can be lower than that of DLP-printed parts^64^. These differences between LCD- and DLP-based printers should be considered based on the available resources and the specific resolution criteria of each application.

In summary, we present an approach for the low-cost and rapid fabrication of biocompatible PDMS microfluidic devices for characterizing drug response, flow-based gradient generators, and cell invasion dynamics in a 3D environment. This approach may be extended to a myriad of other microfluidic bioassays. Using our fabrication process, we demonstrated high biocompatibility for several resins and achieved resolution comparable to that of other technologies for a fraction of the cost. Additionally, we exploited the true 3D design freedom of 3D printing by proposing a novel microrail-based hydrogel-hydrogel interface that would be difficult to fabricate with conventional lithography. We envision that this low-cost setup will facilitate the adoption of microfluidic fabrication by a wider audience. As advancements in vat photopolymerization 3D printing resolution continue^65^, we believe this technology will replace conventional lithography approaches for some cell culture applications by enabling faster and less expensive LCD-based 3D printing.

### Supplementary information

Supplementary Figures
