# Supplementary material for: Fabrication of 3D-printed molds for polydimethylsiloxane-based microfluidic devices using a liquid crystal display-based vat photopolymerization process: printing quality, drug response and 3D invasion cell culture assays
Source: Microsyst Nanoeng. 2023 Nov 9;9:140. doi: 10.1038/s41378-023-00607-y (PMC10632127; doi:10.1038/s41378-023-00607-y)
Supplement: Supplementary file 1 — Supplementary Figures [file 41378_2023_607_MOESM1_ESM.pdf]

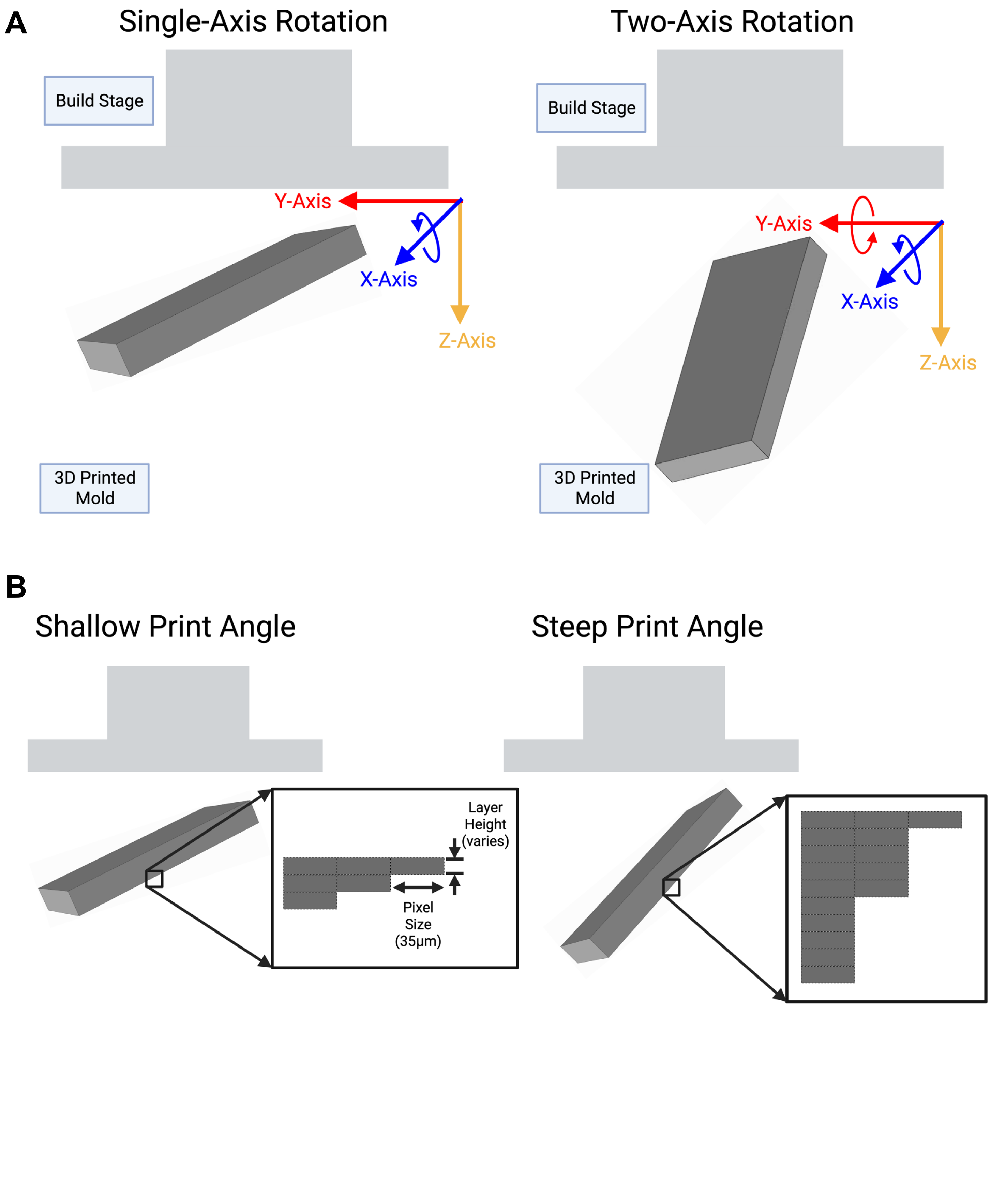

Supplementary Figure 1: Mold printing orientation affects aliasing. (A) Graphical depiction of mold orientation in either single-axis (left) or two-axis (right) rotation during printing. Print angle is the angle of rotation about an axis. (B) Shallower print angle (left) minimizes stairstepping (aliasing) compared to steeper printing angles for a given layer height.

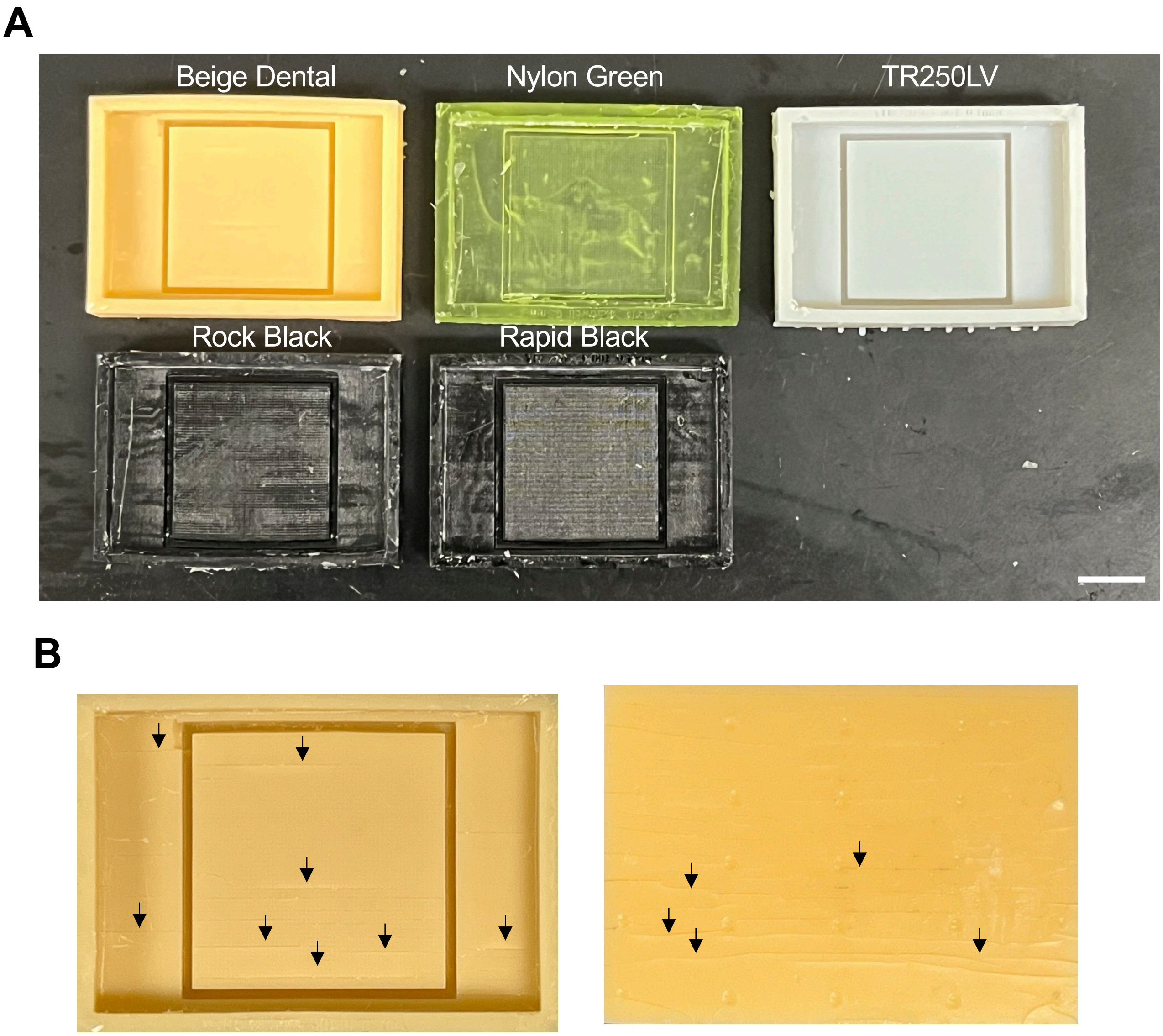

Supplementary Figure 2: Microwell molds after thermal postcure stage. (A) Images of 3D printed molds of five tested resins. Scale bar represents 1cm. (B) Cracks formed in beige dental resin on the top (left) and back (right) of mold after thermal postcure. Black arrows indicate examples of cracks.

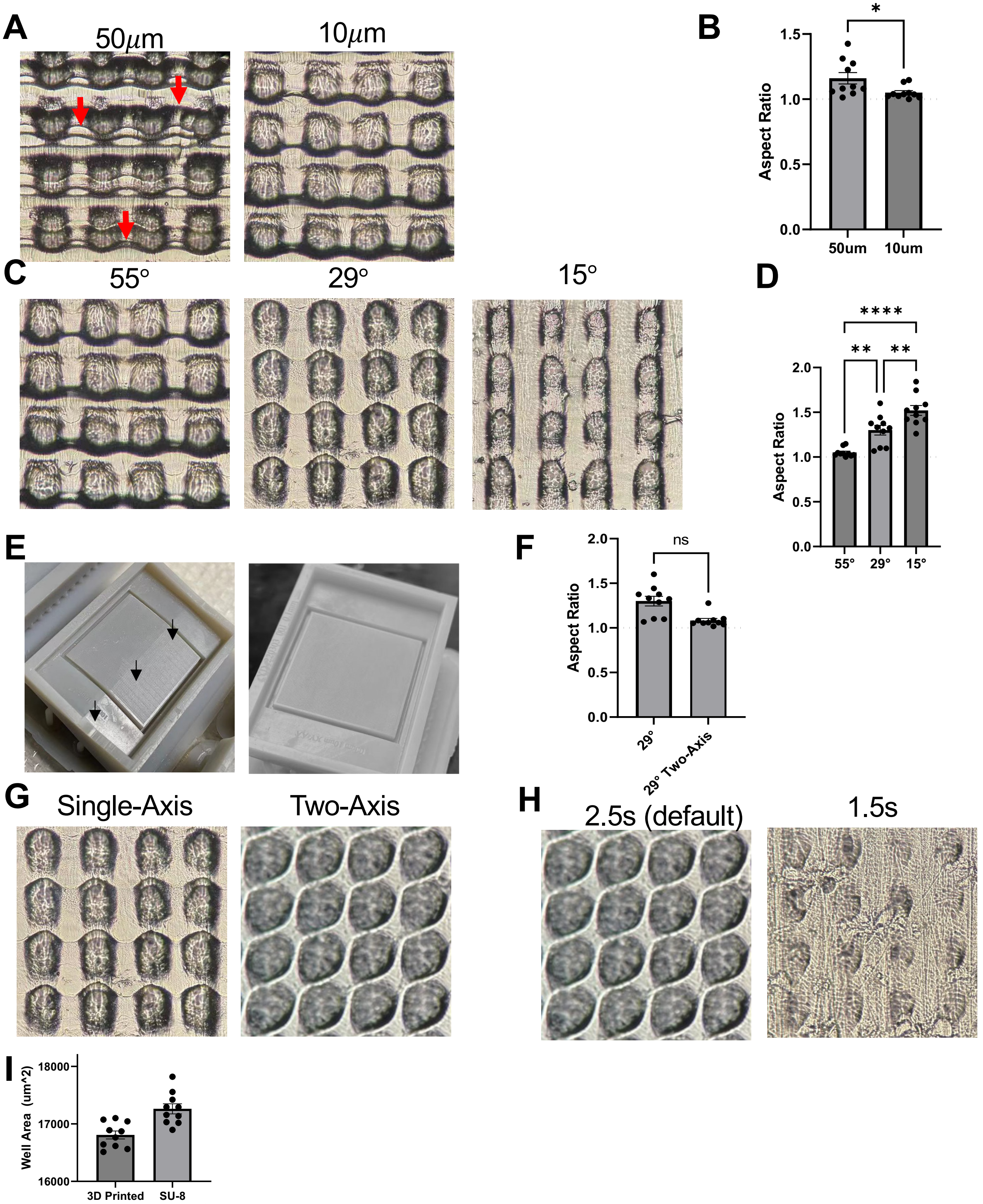

Supplementary Figure 3: Optimization of printing parameters to maximize feature quality. (A) Comparison of PDMS molds at 50μm (left) or 10μm (right) layer height. Red arrows indicate examples of horizontal aliasing lines. (B) Quantification of PDMS microwell aspect ratio. (C) Comparison of PDMS from molds printed at various orientations. Statistical analysis performed using Student's t test. (D) Quantification of aspect ratio for various printing orientations. Statistical analysis was performed using One-Way ANOVA followed by Tukey's multiple comparisons test. (E) Mold deformation during single-axis (left) is corrected by two-axis orientation (right). (F) Quantification of PDMS microwell aspect ratio between single- and two-axis orientation. Statistical analysis performed using Student's t test. (G) Comparison of PDMS from molds printed at either single-axis (left) or two-axis orientation (right). (H) Comparison of PDMS from molds printed at either default exposure time (left) or shortened exposure time (right). (I) PDMS microwell area from 3D printed molds (left) and SU-8 wafer (right). Statistical analysis performed using Student's t test. Error bars denote SEM. Data points representative of 10 randomly selected microwells. *$p<0.05$, **$p<0.01$, ****$p<0.0001$

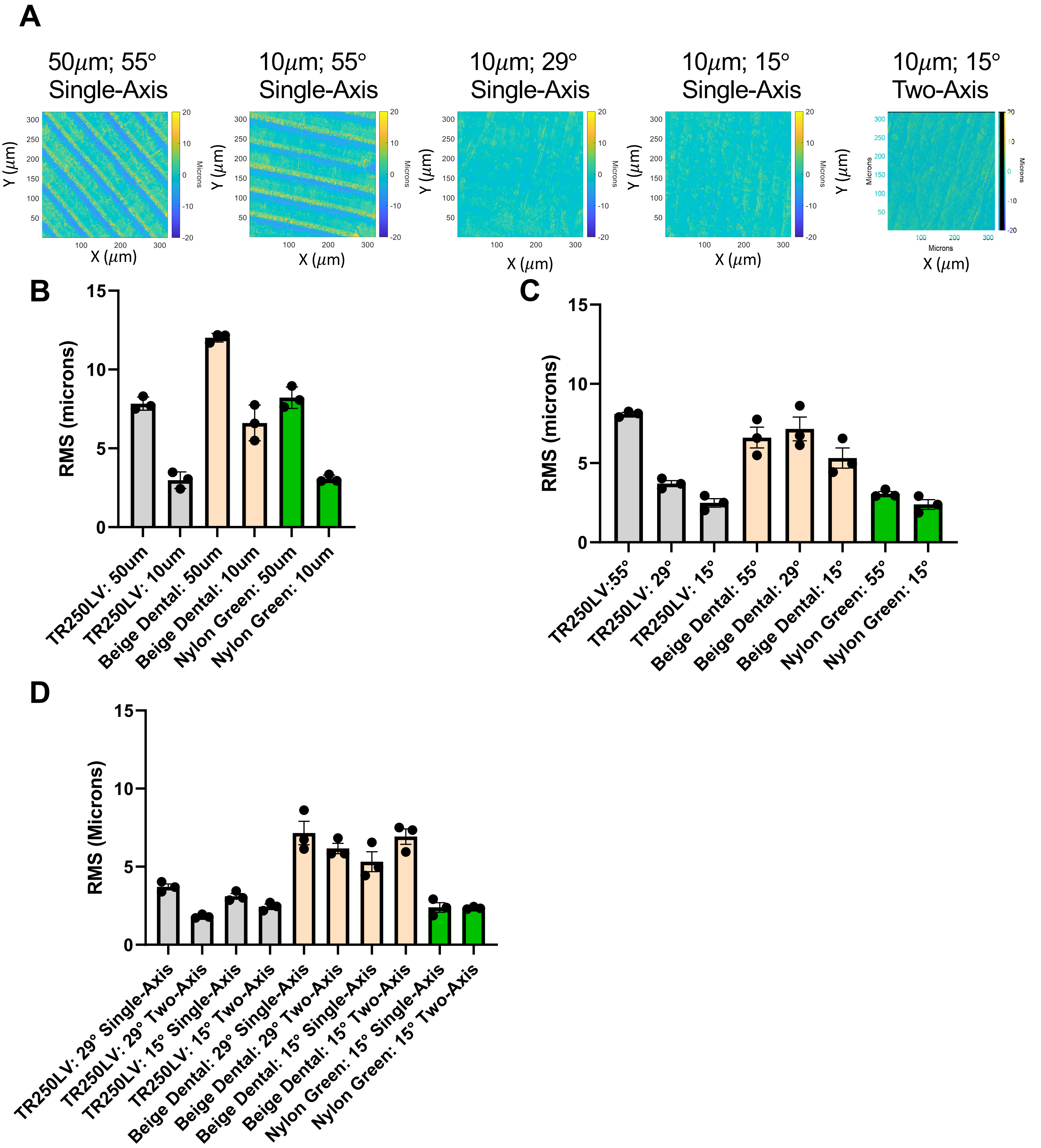

Supplementary Figure 4: Optical profilometry of PDMS from 3D printed molds to characterize surface quality. (A) Representative images of surface of 50μm (left) and 10μm (right) layer height and (B) quantification of surface RMS. (C) Representative images of PDMS from molds printed at 55° (left), 29° (center), and 15° (right) orientations and (D) quantification of surface RMS. (E) Representative images of PDMS from molds printed at 15° orientation on single-axis (left) or two-axis (right) and (F) quantification across three resins. Datapoints represent individual fields captured on one device and are representative of at least two unique molds. Error bars represent SEM.

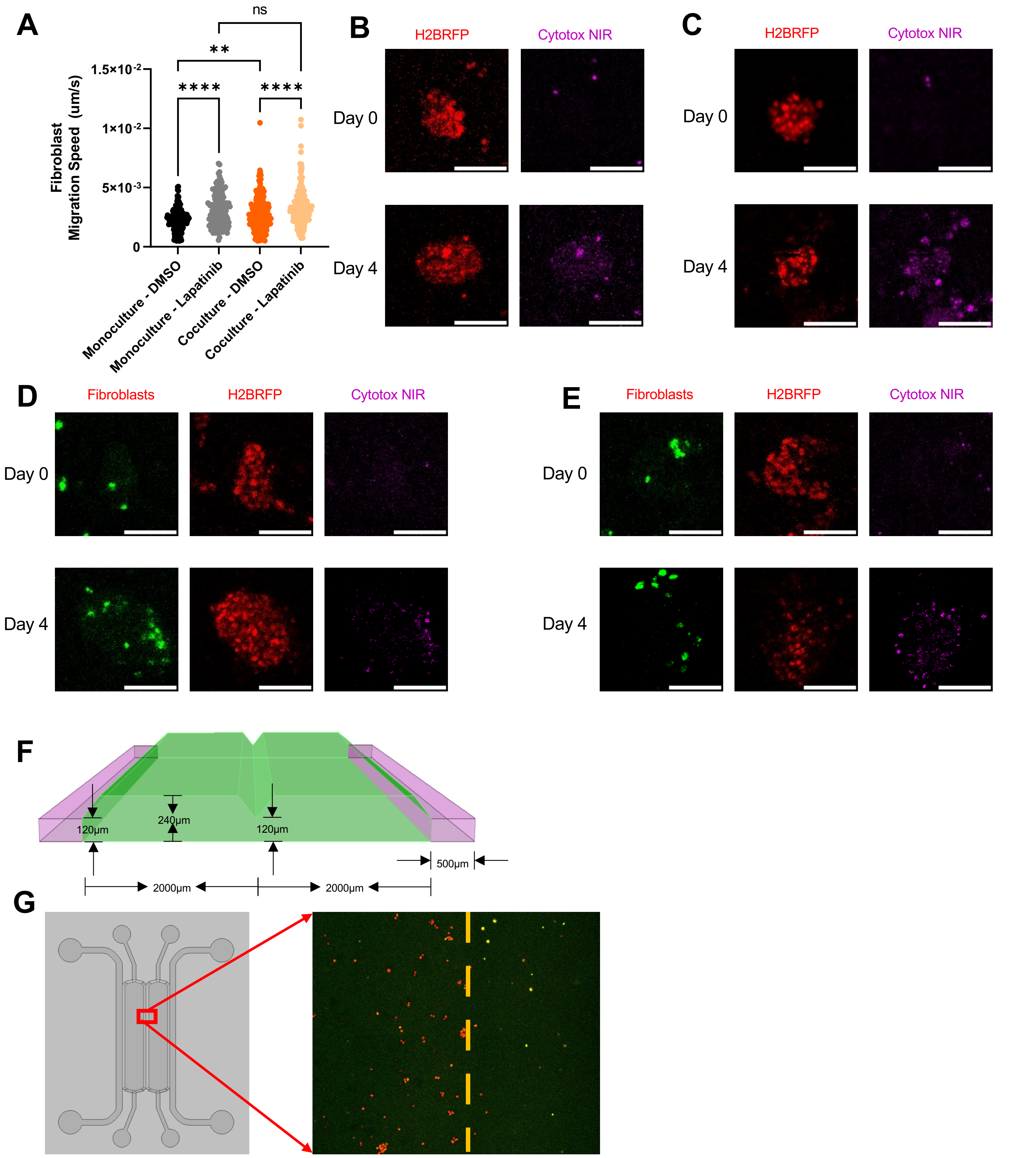

Supplementary Figure 5:Supplementary data for Figure 6. (A) Fibroblast migration speed is increased by lapatinib treatment or coculture with tumor cells. Data is representative of at least two biological replicates with n>400 cells per condition. Statistical analysis was performed using One-Way ANOVA followed by Tukey's multiple comparisons test. Tumor spheroids treated with (B) DMSO or (C) lapatinib after 4 days of treatment. Tumor-fibroblast spheroids treated with (D) DMSO or (E) lapatinib after 4 days of treatment. (F) Novel hydrogel-hydrogel interface to study heterotypic paracrine signaling (G) Representative image of cell spatial patterning. Dashed line indicates interface between tumor cell channel (left of line) and fibroblast channel (right of line). Scale bars represent 100µm.
